# Microglia in Neuronal Circuits

**DOI:** 10.1155/2013/586426

**Published:** 2013-12-22

**Authors:** Long-Jun Wu, Beth Stevens, Shumin Duan, Brian A. MacVicar

**Affiliations:** ^1^Department of Cell Biology and Neuroscience, Rutgers University, Piscataway, NJ 08854, USA; ^2^Children's Hospital Boston, Harvard Medical School, 300 Longwood Avenue, Boston, MA 02115, USA; ^3^Department of Neurobiology, Zhejiang University School of Medicine, 866 Yu Hang Tang Road, Hangzhou, Zhejiang 310058, China; ^4^Department of Psychiatry, University of British Columbia, 2211 Wesbrook Mall, Vancouver, BC, Canada V6T 2B5

## 1. Introduction

Microglia comprise a unique subset of glial cells as the principal brain immune cells and are actively engaged in physiological and pathological brain functions. Unlike other resident neural cells that are of neuroectodermal origin, microglia are of mesodermal origin and invade the neuroepithelium at early embryonic stages. As resident immune response cells, microglia are extremely sensitive to almost any brain disturbance. Therefore, microglia are traditionally recognized for their immune functions during acute brain injury, such as bacterial meningitis, ischemic stroke, and spinal cord injury, as well as chronic neurological disorders, such as Alzheimer's disease, Parkinson's disease, multiple sclerosis, and neuropathic pain. Recently, the role of microglia in neurodevelopment and neural plasticity in the healthy brains has gained tremendous attention. These exciting results raise an intriguing possibility that microglia can integrate into the neuronal circuits in the healthy and diseased brain.

In support of this notion, it is emerging that microglia have remarkably dynamic processes and are frequently interacting with neurons and synaptic elements. Through these interactions, microglia may monitor neuronal/synaptic activities and thus survey the microenvironment in the brain. Indeed, recent studies have apparently shown that microglia function in neuronal circuits by playing diverse roles in neural development, behavior, and pathology in the brain. Therefore, microglia research has changed the way we think about neuronal network/plasticity and increased our understanding of brain diseases associated with abnormal microglia. Contributions to this special issue provide a snapshot of microglial function in the healthy and diseased brain and propose a fundamental role of microglia in neuronal circuits.

## 2. Microglia in the Healthy Brain

The vivid observation of microglia in the healthy brain through *in vivo* imaging in 2005 was a breakthrough in microglia research. For the first time, researchers witnessed that microglia are extremely motile and their processes are constantly monitoring the microenvironment without any pathological insults. Subsequently, studies were booming to focus on the potential role of microglia in the healthy brain, including synaptic pruning in the development and regulation of synaptic transmission/plasticity. On the other hand, several lines of evidence have also indicated the neuronal control of microglial activities under physiological conditions. In this special issue, U. B. Eyo and L. J. Wu highlight recent findings on this bidirectional interaction between neurons and microglia. The review summarized how microglia signal to neurons through direct physical contact or signaling molecules such as fractalkine, compliment, and DAP12, as well as how microglial activity is modulated by neuronal signals including classic neurotransmitters and chemotactic signals. In addition, the authors discussed studies of microglial depletion as an approach to understand microglial importance in neuronal development, function, and maintenance. This review on bi-directional microglial-neuronal communication provides an overview of how microglia are integrated into neuronal circuits in the healthy brain.

Recent studies have revealed a surprising role of microglia in the structural remodeling of neuronal circuits by using their immune abilities in the healthy brain. For example, microglia were demonstrated to eliminate neuronal precursors, synaptic elements, and newborn cells during adult neurogenesis. In this special issue, Z. Šišková and M.-E. Tremblay further zoom in on the microglial function in the neuronal circuits and review recent studies on the microglia-synapse interactions in the mature healthy brain. The focused review discusses the emerging roles of activity-dependent microglial elimination of synaptic elements (dendritic spines and axon terminals) notably by phagocytosis. This microglia-synapse interaction enables synaptic pruning and thus might be crucial for the experience-dependent remodeling of neuronal circuits in the mature brain as well as during normal aging.

In addition to structural remodeling, microglia are able to modulate synaptic activities and plasticity. Evidence from imaging, cellular, and electrophysiological approaches indicates that microglia affect synaptic maturation during development as well as the acute and dynamic regulation of neuronal activity in the mature healthy brain. In this special issue, S. E. Tsirka and colleagues review the recent studies on microglia as an active player in the regulation of synaptic activities and suggest that microglia are an important contributor to the potential quad-partite synapse. The review summarized some interesting mechanisms underlying microglial regulation of synaptic activities and synaptic numbers: the proteases secreted from microglia to remodel extracellular matrix, the release of microvesicles (shed vesicles or ectosomes) derived from microglia, and connexins and large pore channels as a way by which microglia interact directly with neurons. A plethora of potential messengers mediate the communication between microglia and neurons, including cytokines, purines, glutamate, prostaglandins, and nitric oxide. In this special issue, F. Ferrini and Y. De Koninck particularly discuss a unique microglial signaling molecule, brain-derived neurotrophic factor (BDNF), in controlling neuronal excitability in both physiological and pathological conditions.

## 3. Microglia in the Diseased Brain

Resting microglia rapidly transform into an activated state in most pathological processes, including host defense against infectious organisms, autoimmune inflammation, ischemia, trauma, chronic pain, and neurodegeneration. Activation of microglia is accompanied by changes in morphology, upregulation of immune surface antigens, production of cytotoxic or neurotrophic molecules, and phagocytosis of pathogens, degenerating cells, and inflammatory debris. Although microglial activation is well documented in a variety of neurological disorders, the definitive beneficial or detrimental roles of microglia in these diseases remain controversial. The consensus is that microglia play different roles based on the temporal and spatial context of brain diseases; the proinflammatory cytotoxic aspects of activated microglia might be important at an early stage while microglia's anti-inflammatory effects become more prominent later during tissue repair. Nevertheless, microglia evidently respond and even cause the abnormality of neuronal circuits under pathological conditions.

Neuronal cell death, loss of synapses, and neuroinflammation are hallmarks and emerged as a major correlate of cognitive decline in neurodegenerative disorders. In this special issue, Z. Šišková and M.-E. Tremblay extend the discussion of microglia-synapse interaction to the context of neurodegenerative disorders, such as Alzheimer's disease, Parkinson's disease, and prion diseases. Chronic microglial activation under these pathological conditions likely contributes to synaptic dysfunction and elimination, thereby exacerbating neurodegeneration. Richardson and Hossain specifically review recent studies on the role of microglia in Parkinson's disease. Activated microglia and subsequent neuroinflammation have been consistently associated with the pathogenesis of Parkinson's disease. Therefore, the authors discuss the potential of targeting microglia to reduce neuroinflammation, with particular focus on microglial ion channels as novel therapeutic targets for neuroprotection in Parkinson's disease.

The physiology of microglia in the spinal cord is less well studied; however, there is strong evidence of spinal cord microglia in the genesis of chronic pain. In this special issue, R.-R. Ji and colleagues discuss the microglial activation through the mitogen-activated kinase pathways, as well as microglial mediators (tumor necrosis factor-alpha, interleukin-1 beta, and BDNF) in regulating synaptic plasticity of pain circuits in the spinal cord in neuropathic pain. Ferrini and De Koninck focus specifically on microglial BDNF in multiple neurological conditions, including epilepsy, drug addiction, spinal cord injury, and neuropathic pain. In particular, microglial BDNF in the spinal cord is well established in neuronal disinhibition in neuropathic pain in the following signaling cascade: the BDNF activation of neuronal TrkB receptor, downregulation of the K^+^-Cl^−^ cotransporter KCC2, disruption of Cl^−^ homeostasis, and hence the reduced strength of GABA_A_- and glycine receptor-mediated inhibition. Spinal cord injury triggers inflammation with activation of innate immune responses, where both microglia and macrophages are activated and accumulated. In this special issue, Y. Ren and W. Young review the beneficial mechanisms of macrophages on spinal cord injury by inhibition of proinflammatory responses, stimulation of angiogenesis, secretion of neurotrophic factors, and clearance of myelin debris in the injured spinal cord, providing a rationale of macrophage-based therapies for spinal cord injury. Therefore, insights into the communication between microglia/microphages and neurons in the spinal cord will not only further our understanding of microglia function in neuronal network but may also lead to novel therapeutics for ameliorating a wide array of neural dysfunctions, including chronic pain and spinal cord injury.

## 4. Concluding Remarks

This special issue summarizes a broad range of topics on microglia in neuronal circuits in both the healthy and diseased brains, with particular emphasis on bidirectional microglia-neuron communication, microglial remodeling of synapse, microglial regulation of synaptic activities, microglial BDNF signaling, microglia in neurodegeneration such as Parkinson's disease, spinal microglia in neuropathic pain, and macrophages in the spinal cord injury. In spite of the controversy, it is clear that microglia are important and in need of further study in the central nervous system. We hope that papers published in this special issue will serve to increase the scientific knowledge on microglial function in the brain and offer new perspectives on the potential therapeutics targeting microglia/macrophages in various neurological disorders. The past few years have witnessed many important discoveries in the microglia field; however, there is still a long road ahead for exploring the mechanisms underlying microglial function in neuronal circuits at both the molecular and system levels.

*Long-Jun Wu* *Long-Jun Wu* *Beth Stevens* *Beth Stevens* *Shumin Duan* *Shumin Duan* *Brian A. MacVicar* *Brian A. MacVicar*

